# Patient-reported outcomes in transition from high-dose U-100 insulin to human regular U-500 insulin in severely insulin-resistant patients with type 2 diabetes: analysis of a randomized clinical trial

**DOI:** 10.1186/s12955-016-0541-4

**Published:** 2016-09-30

**Authors:** Samaneh Kabul, Robert C. Hood, Ran Duan, Amy M. DeLozier, Julie Settles

**Affiliations:** 1 Eli Lilly and Company, Lilly Corporate Center, Indianapolis, IN 46285 USA; 2 Endocrine Clinic of Southeast Texas, 3030 North Street, Suite 560, Beaumont, TX 77702 USA

**Keywords:** Severe insulin resistance, Type 2 diabetes mellitus, U-500R, Patient compliance, High-dose insulin therapy, Patient-reported outcomes

## Abstract

**Background:**

Initiation and titration of human regular U-500 insulin (U-500R) with a dosing algorithm of either thrice daily (TID) or twice daily (BID) improved glycemic control with fewer injections in patients with type 2 diabetes treated with high-dose, high-volume U-100 insulin. The objective of this analysis was to compare patient-reported outcomes between U-500R TID and BID treatment groups in this titration-to-target randomized, clinical trial.

**Methods:**

In this 24-week, open-label, parallel trial, 325 patients were randomized to TID (*n* = 162) or BID (*n* = 163) U-500R after a 4-week lead-in period (screening). The Treatment Related Impact Measure-Diabetes (TRIM-D) and EQ-5D-5L questionnaires were administered at screening, baseline/randomization, and endpoint (24 weeks). The Visual Analog Scale-Injection Site Pain (VAS-ISP) was assessed at baseline/randomization, 12 weeks, and endpoint.

**Results:**

The TRIM-D showed statistically significant improvements in overall scores from baseline to endpoint for both BID and TID groups, most domains in the TID group, and all domains in the BID group. The BID group achieved better scores than the TID patients in overall and in treatment burden, daily life, and compliance domains (*p* < .05). EQ-5D-5L index scores showed no statistically significant differences for TID and BID groups (and no differences between TID and BID groups) from baseline to endpoint. VAS-ISP scores improved for both treatment groups (−5.60 TID; −6.47 BID; *p* < .05 for both) from baseline to endpoint.

**Conclusions:**

U500 can be successfully titrated for improved glycemic control using BID and TID regimens with diabetes-specific Patient-Reported Outcomes showing improvements in both arms; however, BID had better scores than TID in overall, treatment burden, daily life, and compliance domains.

**Trial registration:**

These secondary analyses are based on the study first received January 22, 2013 and reported in Clinical Trial Registry No.: NCT01774968.

## Background

Severely insulin-resistant patients (daily insulin requirement >200 units or >2 units/kg [1, 2]) with type 2 diabetes treated with high-dose insulin regimens are particularly burdened by longstanding inadequate glycemic control, multiple daily insulin injections, frequent glucose monitoring, obesity, highly prevalent comorbidities, and high healthcare costs, and often have compromised adherence [1–3]. Treatment using high doses of U-100 insulins intensifies barriers to use, as the number of daily injections, injection site discomfort, costs, and impaired adherence also increase [4–7].

Highly concentrated human regular U-500 insulin (U-500R; Humulin^®^ R U-500, Eli Lilly and Company) is a treatment option that may alleviate some of these barriers. For a patient transitioning from a high-dose, high-volume regimen of U-100 insulin (100 units/mL) to one using U-500R, there is a reduction in the volume and the number of daily injections [3], in addition to the potential for decreased costs and improved adherence [4, 7]. While the use of U-500R has also been shown to improve patient satisfaction compared to high-dose U-100 insulins [5, 6] in previous retrospective analyses, this is the first study measuring patient perceptions in a controlled, randomized, clinical trial setting using U-500R.

In the clinical trial, 325 severely insulin-resistant patients with type 2 diabetes on high-dose U-100 insulin (>200 units of insulin/day) with or without oral antihyperglycemic agents were randomized to receive U-500R thrice daily (TID) or twice daily (BID), which was initiated and titrated over a 24-week period in place of U-100 insulins [3]. The objective of this analysis within the primary study was to compare patient-reported outcomes in the form of a diabetes treatment-specific questionnaire (Treatment Related Impact Measure-Diabetes [TRIM-D]), a quality of life questionnaire (EQ-5D-5L), and a pain scale, the Visual Analog Scale-Injection Site Pain (VAS-ISP), before and after initiation of U-500R in TID and BID treatment groups. The hypothesis was that all instruments would improve with treatment on concentrated U-500R given the expected reduction in number of daily injections and insulin volume and anticipated glycated hemoglobin (HbA1c) improvement.

## Methods

### Procedures

The detailed trial design, including a description of the study population, has been previously reported but is discussed here briefly [3]. Baseline demographic and clinical characteristics of the TID and BID patients are provided in Table 1. For the overall study population, baseline body mass index was 41.9 ± 7.5 kg/m^2^, HbA1c was 8.7 % ± 1.0 %, median number of daily injections was 5 (range 2–10), and total daily dose (TDD) was 287.5 ± 80.5 units/day (2.4 ± 0.8 units/kg/day) [3]. Baseline insulin therapies included basal bolus (69.6 % [96.5 % analog insulins]), premixed insulin (12.3 %), basal only (6.2 %), and other (12.0 %) [3]. All patients were prior U-100 insulin users who were placed on a 4-week lead-in period, during which U-100 doses were verified and adjusted per investigators’ judgment. Patients were then randomized to receive subcutaneous U-500R TID (*n* = 162) or BID (*n* = 163) [3]. The syringes provided for administration were 6-mm, 31-gauge U-100 insulin syringes (Becton, Dickinson and Company) and dosing was recommended 30 min before meals for both treatment groups. Patient-reported outcome measures, including the TRIM-D [8], EQ-5D-5L [9, 10], and VAS-ISP [11], were used to compare changes in diabetes treatment-related impact measures, quality of life, and injection site pain from baseline to endpoint between and within the BID and TID treatment groups. The TRIM-D and EQ-5D-5L assessments were performed at visit 1 (screening, -4 weeks), visit 3 (baseline/randomization, 0 weeks), and visit 16 (endpoint, 24 weeks) or early termination (if patients did not reach 24 weeks of study). The VAS-ISP was completed at visit 3 (baseline/randomization), visit 12 (12 weeks), and visit 16 (endpoint). Descriptive statistics by treatment and injection method were also provided as part of this analysis. The ethics review boards provided written approval of the study protocol and the informed consent form. The study was conducted in accordance with the International Conference on Harmonisation Guidelines for Good Clinical Practice and the Declaration of Helsinki and all patients provided written informed consent.

**Table 1:** Baseline demographics and clinical characteristics of patients randomized to the TID and BID dosing algorithms^a^

	U500R TID (*n* = 162)	U500R BID (*n* = 163)	*p* value
Male, n (%)	83 (51.2)	89 (54.6)	.54
Race, n (%)			.66^b^
White	133 (82.1)	133 (81.6)	
Black	21 (13.0)	19 (11.7)	
Asian	2 (1.2)	4 (2.5)	
Native American	3 (1.9)	1 (0.6)	
Other	3 (1.9)	6 (3.7)	
Ethnicity (Hispanic), n (%)	32 (19.8)	30 (18.4)	.76
Age, years^c^	55.3 ± 10.5	55.5 ± 9.0	.86
Weight, kg	120.9 ± 25.1	122.9 ± 26.2	.48
BMI, kg/m^2^	41.8 ± 7.6	41.9 ± 7.3	.95
Baseline HbA1_c_	8.7 ± 1.1	8.7 ± 1.0	.87
Diabetes duration, years	14.9 ± 6.8	15.5 ± 8.0	.51
TDD, (U-100, final), units	287.1 ± 79.9	287.8 ± 81.2	.94
Number of injections, median [min, max]	5 [2, 9]	5 [2, 10]	.96

*Abbreviations*: *BID* twice daily; *BMI* body mass index; *HbA1*_*c*_ glycated hemoglobin; *TDD* total daily dose; *TID* thrice daily

*p* values for continuous variables are based on analysis of variance, and for categorical variables, Fisher’s exact or chi-square test

^a^Excerpted from reference [3] Hood RC, et al. 2015

^b^Combined values

^c^Values are presented as means ± SD unless otherwise noted

### Measures

The TRIM-D is a validated, patient-reported outcome measure that assesses treatment-related impact on participants with type 1 or type 2 diabetes [8, 12, 13]. The instrument consists of 28 items that are measured on a 5-point Likert scale, with higher scores indicating a better health state. TRIM-D items make up the following five domains: treatment burden (six items), daily life (five items), diabetes management (five items), compliance (four items), and psychological health (eight items). For example, items on the compliance domain depict how often a medication is missed, delayed, or postponed on a response scale ranging from never/almost never to almost always/always. Transformed scale scores range from 0 to 100, with higher scores indicating a better health state (less negative impact). The reliability and validity for TRIM-D has been previously assessed [12].

The EQ-5D-5L is a generic, well-validated, patient-reported outcome measure consisting of the descriptive system and Visual Analog Scale (EQ-5D VAS) [9, 10]. The descriptive system captures 5 dimensions (mobility, self-care, usual activity, pain/discomfort, and anxiety/depression), and each dimension has 5 levels (no problems, slight problems, moderate problems, severe problems, and extreme problems). The respondents were asked to indicate their health status by checking item boxes on the questionnaire that fit the way they felt about their health on that day. The EQ-5D VAS measures the respondent’s self-rated health on a 20-cm vertical VAS numbered from 0 to 100, with 0 representing the worst health and 100 the best health imaginable. The EQ-5D-5L has been validated in a diverse patient population in 6 countries and many different chronic disease states, including diabetes, for all dimensions and all levels [10]. Reliability and responsiveness remain to be assessed for the EQ-5D-5L [10].

The VAS-ISP assesses injection site pain over the past 24 h using a 100-mm horizontal VAS with anchors from “no pain” to “as severe as I can imagine” derived from Huskisson [11]. Early in the study, the VAS-ISP version of the questionnaire administered in this trial was modified from pain intensity ‘now’ to a corrected version of the VAS-ISP questionnaire with the time frame of the ‘past 24 h’. Results from both instruments were analyzed and found to be comparable. The reliability and responsiveness of the VAS-ISP has not been assessed.

### Statistical analyses

Baseline measurements for continuous variables were evaluated using analysis of variance. The EQ-5D-5L US Index and EQ-5D VAS values were calculated using the crosswalk method [10]. The TRIM-D, EQ-5D-5L, and VAS-ISP scores were compared between treatment groups at screening and baseline using analysis of covariance (ANCOVA). Change from baseline to endpoint scores were compared between treatment groups using the ANCOVA model with treatment as factor, baseline HbA1c, baseline TDD, investigator site, and baseline score as covariates. Comparisons of within-treatment group scores were conducted using mixed-effect model repeated measures (MMRM) with treatment, visit, interaction between treatment and visit, baseline HbA1c, TDD, investigator site, and baseline score as covariates using an unstructured covariance matrix.

The study population was categorized based on reduction of per-day insulin injections in the transition from U-100 to U-500 (0–2, 3–4, >4 per day). TRIM-D and EQ-5D-5L scores for subgroups were compared between treatment groups using an ANCOVA model. Based on the conventional definition of 80 % compliance being clinically meaningful for adherence to diabetes medications, an arbitrary cutoff of TRIM-D compliance score ≥80 (the lowest and highest possible raw scores are 28 and 140, and after transformation, the lowest and highest possible score is 0–100) was used to compare the percentage achieving scores of ≥80 versus <80 between the two treatment groups [14, 15]. Logistic regression was used with treatment, baseline score, and baseline stratification variables (baseline HbA1c ≤8 % or >8 %, baseline TDD ≤300 or >300 units) as independent variables. The dependent variable was patients achieving a TRIM-D score ≥80.

The sample size calculation was based on the primary outcome: change in HbA1c from randomization to week 24 [3]. It was estimated that 260 completers would provide at least 80 % probability to determine non-inferiority of BID to TID, or vice versa, for the primary endpoint (non-inferiority margin 0.4 %). Patient-reported outcomes were exploratory. The *p*-values < .05 were considered statistically significant. All analyses were performed using SAS version 9.2 or higher (SAS Institute, Inc., Cary, NC) and were based on the full analysis set.

## Results

Baseline demographic and clinical characteristics of patients titrated to the TID and BID algorithms were well balanced (Table 1). The two treatment groups were comparable at baseline with no significant differences in these measured variables.

### TRIM-D

Patients within each treatment group had statistically significant improvement overall and within each TRIM-D domain (treatment burden, daily life [BID only], diabetes management, compliance, and psychological health) from baseline to endpoint (Table 2). Individuals in the BID group achieved statistically significant improved scores overall, and in the treatment burden, daily life, and compliance domains when compared to those in the TID group at endpoint.

**Table 2:** Comparisons of TID and BID dosing algorithms using TRIM-D patient-reported outcomes

TRIM-D Domain	U-500R Treatment (n)	Mean Actual Value (SD)	Change from Baseline (MMRM) LSM (95 % CI)^a^	LSM Difference between Treatments (95 % CI) vs. U-500R TID^b^
Overall				
Screening	TID (161)	60.28 (13.61)	−2.65 (−4.77,−0.53)*	0.50 (−2.57, 3.56)
	BID (159)	60.77 (14.27)	−2.53 (−4.67,−0.38)*	
Baseline	TID (161)	61.35 (14.59)		1.09 (−2.05, 4.22)
	BID (161)	62.44 (14.00)		
Endpoint	TID (159)	68.31 (14.14)	6.04 (3.81, 8.26)**	3.52 (0.93, 6.11)*
	BID (153)	72.44 (12.57)	10.06 (7.82, 12.30)**	
Treatment burden				
Screening	TID (160)	60.37 (18.40)	−2.75 (−6.12, 0.63)	−1.23 (−5.57, 3.11)
	BID (159)	59.15 (20.91)	−2.63 (−6.01, 0.75)	
Baseline	TID (160)	62.69 (19.39)	−	−2.48 (−6.93, 1.97)
	BID (160)	60.21 (21.02)		
Endpoint	TID (158)	68.91 (19.31)	6.23 (2.70, 9.75)*	5.03 (0.99, 9.06)*
	BID (153)	72.97 (19.84)	12.24 (8.71, 15.77)**	
Daily life				
Screening	TID (161)	63.72 (17.38)	−0.72 (−3.77, 2.32)	−0.50 (−4.52, 3.52)
	BID (158)	63.22 (19.10)	−1.46 (−4.53, 1.62)	
Baseline	TID (161)	62.50 (17.69)	−	0.97 (−2.95, 4.89)
	BID (161)	63.47 (18.07)		
Endpoint	TID (159)	66.17 (17.78)	1.75 (−1.44, 4.93)	4.01 (0.41, 7.62)*
	BID (153)	70.63 (17.57)	6.58 (3.37, 9.79)**	
Diabetes management				
Screening	TID (161)	40.18 (19.53)	−3.71 (−7.07,−0.35)*	4.27 (−0.15, 8.69)
	BID (158)	44.45 (20.62)	0.13 (−3.26, 3.52)	
Baseline	TID (161)	43.15 (20.83)	−	0.91 (−3.67, 5.49)
	BID (161)	44.07 (20.95)		
Endpoint	TID (159)	57.02 (18.92)	13.40 (9.89, 16.92)**	0.52 (−3.62, 4.65)
	BID (153)	57.87 (19.75)	14.89 (11.35, 18.43)**	
Compliance				
Screening	TID (161)	65.06 (17.34)	−3.04 (−5.79,−0.29)*	1.24 (−2.64, 5.11)
	BID (158)	66.30 (17.84)	−2.58 (−5.37, 0.22)	
Baseline	TID (161)	67.43 (17.09)	−	2.02 (−1.76, 5.79)
	BID (161)	69.45 (17.33)		
Endpoint	TID (159)	73.00 (16.87)	5.31 (2.43, 8.19)**	4.48 (1.50, 7.47)*
	BID (153)	78.12 (14.52)	9.89 (6.98, 12.80)**	
Psychological health				
Screening	TID (161)	68.09 (19.17)	−2.90 (−5.67,−0.13)*	−0.14 (−4.36, 4.08)
	BID (159)	67.95 (19.20)	−5.00 (−7.80,−2.20)**	
Baseline	TID (161)	68.02 (19.07)	−	3.49 (−0.50, 7.47)
	BID (161)	71.51 (17.23)		
Endpoint	TID (159)	73.90 (19.86)	4.57 (1.66, 7.47)*	3.22 (−0.11, 6.56)
	BID (153)	79.49 (16.00)	7.50 (4.58, 10.43)**	

*Abbreviations*: *BID* twice daily; *CI* confidence interval; *LSM* least-square mean; *MMRM* mixed model for repeated measures; *SD* standard deviation; *TID* thrice daily; *TRIM-D* Treatment Related Impact Measure-Diabetes; *U-500R* human regular U-500 insulin

^a^ *p*-values and 95 % CI of difference of LSM of change from baseline were from the MMRM model

^b^ *p*-values and 95 % CI of difference of LSM of change from baseline were from the ANCOVA model

*p < .05; **p < .001

The median number of U-100 injections per day at baseline was five for both groups, with a range of 2 to 9 for TID and 2 to 10 for BID (Table 1) [3]. Patients were grouped by number of injections reduced from baseline into three categories (0–2, 3–4, and >4), and these subgroups were analyzed separately for TRIM-D overall and specific domains at endpoint (Table 3). Individuals in the U-500R BID group reported significant improvements in overall score and each domain when 0–2 and 3–4 injections were reduced from baseline. The individuals in the TID group reported significant improvements in overall and in each domain when 0–2 and 3–4 injections were reduced from baseline (*p* < .05) except the daily life domain when 0–2 injections were reduced from baseline. Significant differences may not have been seen in the >4 injections reduced category because of the small sample size (two in the TID group and ten in the BID group).

**Table 3:** Relationship between TRIM-D overall and domain scores and the number of reduced injections from baseline to endpoint

TRIM-D Domain	Reduced Number of Injections	U-500R Treatment (n)	Mean Value (SD)	Change from Baseline	LSM Change from Baseline (SE)
Overall	0–2	TID (121)	67.78 (14.60)	5.74 (13.98)	6.15 (1.08)*
		BID (58)	72.73 (12.56)	7.95 (13.37)	10.51 (1.60)*
	3–4	TID (36)	70.54 (12.39)	11.32 (15.78)	8.48 (2.02)*
		BID (85)	72.06 (12.89)	10.83 (13.71)	9.86 (1.29)*
	>4	TID (2)	59.82 (16.41)	3.57 (6.31)	−1.22 (8.31)
		BID (10)	74.02 (10.65)	10.27 (10.88)	9.89 (3.79)*
Treatment burden	0–2	TID (120)	68.65 (19.77)	5.36 (21.62)	7.09 (1.69)*
		BID (58)	70.62 (21.93)	9.55 (25.43)	10.84 (2.47)*
	3–4	TID (36)	70.37 (18.06)	8.91 (21.92)	6.61 (3.14)*
		BID (85)	74.59 (18.91)	15.08 (21.02)	12.78 (2.00)*
	>4	TID (2)	58.33 (17.68)	0.00 (11.79)	−3.42 (12.90)
		BID (10)	72.92 (14.33)	9.58 (19.35)	10.17 (5.89)
Daily life	0–2	TID (121)	65.51 (18.76)	2.24 (19.05)	2.61 (1.50)
		BID (58)	72.52 (17.34)	7.61 (19.99)	8.85 (2.22)*
	3–4	TID (36)	68.89 (14.40)	9.17 (17.42)	6.75 (2.81)*
		BID (85)	69.24 (17.33)	7.18 (19.02)	6.68 (1.79)*
	>4	TID (2)	57.50 (3.54)	−7.50 (10.61)	−8.59 (11.54)
		BID (10)	71.50 (21.61)	4.00 (16.12)	5.46 (5.27)
Diabetes management	0–2	TID (121)	56.46 (19.32)	12.33 (23.57)	12.95 (1.72)*
		BID (58)	57.74 (20.00)	9.63 (24.48)	14.09 (2.55)*
	3–4	TID (36)	59.44 (17.92)	19.83 (27.95)	15.08 (3.23)*
		BID (85)	58.18 (20.15)	15.29 (25.55)	13.87 (2.06)*
	>4	TID (2)	47.50 (10.61)	12.50 (17.68)	5.38 (13.29)
		BID (10)	56.00 (16.30)	15.00 (16.83)	12.38 (6.06)*
Compliance	0–2	TID (121)	72.47 (17.61)	4.17 (15.47)	4.27 (1.24)*
		BID (58)	77.51 (15.30)	8.76 (14.30)	9.77 (1.84)*
	3–4	TID (36)	75.00 (14.56)	9.72 (22.43)	7.34 (2.33)*
		BID (85)	78.09 (14.26)	9.12 (14.96)	9.16 (1.48)*
	>4	TID (2)	68.75 (8.84)	3.13 (13.26)	0.13 (9.58)
		BID (10)	81.88 (12.66)	7.50 (13.44)	9.27 (4.37)*
Psychological health	0–2	TID (121)	73.30 (20.56)	4.76 (17.85)	4.45 (1.38)*
		BID (58)	81.51 (15.17)	5.60 (13.69)	9.39 (2.06)*
	3–4	TID (36)	76.39 (17.12)	9.98 (15.79)	7.53 (2.59)*
		BID (85)	77.61 (16.86)	8.01 (18.10)	7.28 (1.65)*
	>4	TID (2)	65.63 (30.94)	7.81 (2.21)	0.78 (10.68)
		BID (10)	83.75 (11.39)	13.13 (13.24)	11.32 (4.86)*

*Abbreviations*: *BID* twice daily; *LSM* least-square mean; *SD* standard deviation; *SE* standard error; *TID* thrice daily; *TRIM-D* Treatment Related Impact Measure-Diabetes; *U-500R* human regular U-500 insulin

*p < .05; *p*-value and LSM of change from baseline were from an ANCOVA model

The compliance domain scores in the BID treatment group were significantly higher compared to the TID treatment group (*p* = .03 in favor of BID vs. TID). The proportion of patients achieving a TRIM-D compliance score ≥80 was analyzed by subordinate analysis using logistic regression and 36 % (58/159) of patients in the TID treatment group vs. 49 % (75/153) in the BID group had a TRIM-D compliance score of ≥80 (odds ratio = 1.70; 95 % confidence interval: 1.05–2.75; *p* = .03) (Table 4).

**Table 4:** Subanalysis of individuals with TRIM-D compliance score ≥80 at endpoint^a^

	U-500R TID (*n* = 159) n (%)	U-500R BID (*n* = 153) n (%)	Total (*N* = 312) n (%)	Odds Ratio U-500R BID/TID	95 % CI of Odds Ratio	*p*-value
Number of patients with TRIM-D compliance score ≥80	58 (36.5)	75 (49.0)	133 (42.6)	1.70	1.05, 2.75	.03

*Abbreviations*: *BID* twice daily; *CI* confidence interval; *n* number of patients in a specified group; *N* total number of patients; *TID* thrice daily; *TRIM-D* Treatment Related Impact Measure-Diabetes; *U-500R* human regular U-500 insulin

^a^CI and *p*-values were from a logistic regression model with fixed effects for treatment, baseline score and baseline stratification variables (Baseline A1C (≤8 % or >8 %), Baseline TDD (≤300 or >300), and pooled investigator). *p*-value from this analysis was based on a null hypothesis that the odds ratio is one﻿

### EQ-5D-5L

EQ-5D-5L index scores showed no statistically significant differences between the TID and BID groups at screening, baseline/randomization, and endpoint (Table 5). Visit comparisons of total score for the overall population and TID and BID groups were conducted separately. No significant visit differences were found in scores between screening and baseline or between baseline and endpoint.

**Table 5:** EQ-5D-5L index score comparing 2 U-500R dosing algorithms in individuals with severely insulin-resistant type 2 diabetes

EQ-5D-5L US Index - Visit	U-500R Treatment (n)	LSM (SE)	*p-*value Visit Comparison (Change from Baseline)^a^	LSM Difference (95 % CI) vs. U-500R TID
Screening	TID (161)	0.77 (0.01)	0.97	0.01 (−0.02, 0.04)
	BID (158)	0.78 (0.01)	0.35	
Baseline	TID (162)		–	0.00 (−0.03, 0.03)
	BID (161)		–	
Endpoint	TID (159)	−0.02 (0.01)	0.30	0.01 (−0.02, 0.03)
	BID (152)	−0.01 (0.01)	0.80	

*Abbreviations: BID* twice daily; *CI* confidence interval; *LSM* least-square mean; *SE* standard error; *TID* thrice daily; *U-500R* human regular U-500 insulin

^**a**^ *p*-values and 95 % CI of difference of LSM of change from baseline were from the MMRM model

Although the number of injections in the TID and BID cohorts declined during the transition period of the study [3], neither the treatment (TID or BID) nor the number of reduced injections (i.e., 0–2, 3–4, or >4) had significant effect on the EQ-5D-5L index scores.

Summaries of responses which best described the respondent’s health on the five EQ-5D-5L domains (mobility, self-care, usual activities, pain/discomfort, anxiety/depression) showed no statistically significant difference between treatment groups at screening, baseline, or endpoint.

Quantitative EQ-5D VAS scores for overall health at screening, baseline, and endpoint in TID respondents were 70.0, 70.4, and 68.0, respectively (TID endpoint least-square means *p* < .05 compared to baseline); whereas, scores in BID respondents were not significantly different at 68.9, 70.8, and 70.0. Comparisons between TID and BID treatment groups were not significantly different.

### VAS-ISP

**Fig. 1:**
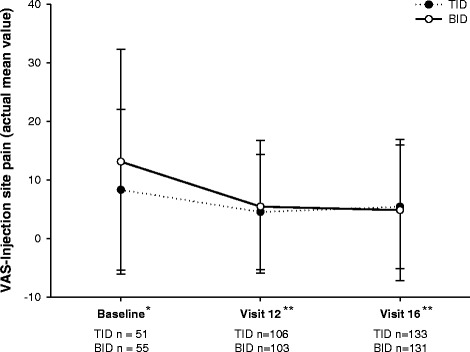
Visual Analog Scale - Injection Site Pain evaluation from baseline to endpoint. Abbreviations: BID = twice daily; SD = standard deviation; TID = thrice daily; VAS = visual analog scale. *Actual mean values of patients on U100 insulins ± SD are from corrected CRF data. **p-values are <0.05 for visit 12 and visit 16 when least square mean change from baseline are calculated (−7.36 TID, -5.85 BID visit 12 and -5.60 TID, -6.47 BID visit 16)

VAS-ISP demonstrated a significant improvement in injection site pain from baseline to endpoint for TID and BID treatment groups (least square means change from baseline at visit 12, −7.36 TID; −5.85 BID; and at visit 16, −5.60 TID; −6.47 BID; *p* < .05; Fig. 1). No significant difference of score reduction between BID and TID groups was evident.

## Discussion

This is the first analysis of prospective and comprehensive patient-reported outcomes from a randomized, clinical trial of severely insulin-resistant patients with type 2 diabetes who were switched from high-dose U-100 insulin regimens to U-500R as insulin monotherapy. Consistent with the initial study hypothesis, transitioning from high-dose, high-volume U-100 insulin requiring multiple daily injections and implementing TID or BID U-500R dosing algorithms, most TRIM-D domain scores in patients demonstrated clinically relevant and significant improvements from baseline to endpoint. By endpoint, respondents in the BID group had experienced greater perceived improvements in TRIM-D over time when compared to the TID group. Respondents also reported improved compliance from baseline for both groups, and a higher proportion of BID group patients achieved a TRIM-D compliance score ≥80.

As first reported by Hood et al. [3], the primary study outcome, change in HbA1c between TID and BID, was clinically equivalent between the two study groups (−1.12 % TID;−1.22 % BID; difference,−0.10 %; 95 % confidence interval:−0.33 to 0.12 %; noninferiority margin, 0.4 %). Despite an increase in TDD and significant reduction in HbA1c [3], both groups reported an improvement in compliance scores. This could be explained by better adherence with simplified insulin regimens wherein up to 10 injections daily of U-100 were prescribed before randomization (often with more than one insulin), compared to a monotherapy regimen of two or three injections of U-500R post-randomization.

Although EQ-5D VAS comparisons between the TID and BID groups were not significantly different, individuals in the TID group responded with statistically lower health scores from baseline at endpoint. Because the EQ-5D-5L is not diabetes specific, the assessment may not have been sensitive enough to distinguish between treatment groups.

Injection site pain was significantly reduced in both treatment groups, which might be attributed to the reduced daily injection volume (−2.2 mL for TID and BID) and reduced number of daily injections (−2 TID,−3 BID) [3, 16]. This finding supports prior reports of reduced injection site discomfort with use of concentrated U-500R [5, 17–19].

Limitations of this study included the open-label design and lack of direct measures of compliance. Patient perceptions of use while on vial and syringe versus pen regimens prior to randomization to U-500R were not evaluated separately, which could also have impacted patient perception of the new insulin regimen (vial and syringe).

Since this analysis takes into account the patient perspective, an additional factor not analyzed here that could affect the patient perception is the affordability of insulin. In recent years, this has become a significant issue, especially for those patients requiring high doses of insulin [20] to achieve glycemic control. For the majority of patients in this trial using basal + bolus analog insulin regimens (67 % of patients) prior to randomization to U-500R, one would expect that the switch to U-500R would lead to a reduction in daily insulin costs. This decrease might be expected to occur after accounting for an increase in TDD from baseline to endpoint regimens after 24 weeks as part of a titration-to-target intensification. While this reduction in real-world drug costs with transition to U-500R has previously been reported as compared to propensity-matched patients remaining on high-dose conventional U-100 insulins [4, 7], further detailed economic analyses on this topic would be of value to patients, payers, and providers alike.

## Conclusion

Our study findings indicate that initiation and titration of U-500R leads to improved patient perception, improved impact of diabetes treatment on functioning and well-being, and less injection site discomfort following use of high-dose, high-volume U-100 insulins in severely insulin-resistant patients with type 2 diabetes. While both regimens improved patient reported treatment-related diabetes measures, the twice-daily regimen showed more of an improvement in certain patient reported domains when compared to the thrice-daily regimen. Its impact on compliance, specifically, should therefore be considered when choosing an appropriate U-500 regimen in a clinical practice setting. These results are complementary to the primary clinical trial findings, which showed significantly improved glycemic control with low incidence and rates of severe hypoglycemia with fewer daily injections [3]. These results can be further complemented by appropriately designed, prospective, real-world analyses accounting for other patient and economic factors that were not studied here.

## Abbreviations

ANCOVA: analysis of covariance
BID: twice daily
EQ-5D-5L: quality of life questionnaire
HbA1c: glycated hemoglobin
MMRM: mixed-effect model repeated measures
TDD: total daily dose
TID: thrice daily
TRIM-D: Treatment Related Impact Measure-Diabetes
U-500R: human regular U-500 insulin
VAS: visual analog scale
VAS-ISP: Visual Analog Scale-Injection Site Pain
